# Spx mediates oxidative stress regulation of the methionine sulfoxide reductases operon in *Bacillus subtilis*

**DOI:** 10.1186/1471-2180-8-128

**Published:** 2008-07-28

**Authors:** CongHui You, Agnieszka Sekowska, Olivera Francetic, Isabelle Martin-Verstraete, YiPing Wang, Antoine Danchin

**Affiliations:** 1 Institut Pasteur, Unité de Génétique des Génomes Bactériens, 28 rue du Docteur Roux, 75724 Paris Cedex 15, France; 2 National Laboratory of Protein Engineering and Plant Genetic Engineering, The College of Life Sciences, Peking University, 100871, Beijing, PR China; 3 Institut Pasteur, Unité de Génétique Moléculaire, 25 rue du Docteur Roux, 75724 Paris Cedex 15, France; 4 Department of Physics and Center for Theoretical Biological Physics, University of California at San Diego, La Jolla, CA 92093-0374, USA

## Abstract

**Background:**

All aerobically grown living cells are exposed to oxidative damage by reactive oxygen species (ROS). A major damage by ROS to proteins is caused by covalent modifications of methionine residues giving methionine sulfoxide (Met-SO). Methionine sulfoxide reductases are enzymes able to regenerate methionine and restore protein function after oxidative damage.

**Results:**

We characterized the methionine sulfoxide reductase genes *msrA *and *msrB *in *Bacillus subtilis*, forming an operon transcribed from a single sigma A-dependent promoter. The *msrAB *operon was specifically induced by oxidative stress caused by paraquat (PQ) but not by H_2_O_2_. Spx, a global oxidative stress regulator in *B. subtilis*, is primarily responsible for this PQ-specific induction of *msrAB *expression. In support of this finding, an *spx *deletion mutant is extremely sensitive to PQ, and increased expression of *msrA *was identified in a *clpX *mutant in which Spx accumulated. However, the Spx effect was also visible under conditions where the protein did not accumulate (PQ treatment), suggesting a specific molecular effect at the level of the Spx protein. Indeed, the CXXC motif of Spx was found essential for its function in the PQ-specific induction of *msrAB *expression. PQ caused a modification of Spx requiring at least one of the cysteines of the CXXC motif of Spx. The PQ modified form of Spx showed a dynamic change *in vivo*.

**Conclusion:**

The Spx mediated PQ-specific regulation pathway of the *msrAB *operon in *B. subtilis *is reported. Our results suggest that PQ induced the expression of *msrAB *partially through an oxidation on Spx via modification of its CXXC motif.

## Background

Among the 20 protein-building amino acids, methionine has a special status. It is the first amino acid in all nascent peptides, and often retained in the mature polypeptides. Methionine residues in proteins are also involved in catalytic centers [1-5] and binding of metals, copper in particular [6,7]. However, its major role in the cell is sometimes attributed to the reactivity of its sulfur atom. Methionine is highly sensitive to reactive oxygen species (ROS) that modify it covalently, yielding methionine sulfoxide in two enantiomeric forms: *R*- and *S*-. Remarkably, this reaction is reversible and two non-homologous methionine sulfoxide reductases MsrA and MsrB can restore intact methionines from the *S*- and *R*- forms, respectively (reviewed in [8]). Despite a long interest in this modification and repair, we still lack much understanding about the control of the cognate regulation pathway. A series of thorough analyses have been performed in Enterobacteria (*Escherichia coli *[9] and *Erwinia chrysanthemi *[10]), but our knowledge is still scarce in Gram-positive organisms, despite the obvious importance of ROS in pathogenicity [11].

In the genome of *B. subtilis*, the two methionine sulfoxide reductase genes, *msrA *and *msrB *(*yppQ*), are adjacent in the chromosome and probably form an operon [12]. The function of these enzymes and the regulation of their synthesis have not been studied. The only experimental report related to methionine sulfoxide reductase in *B. subtilis *is the demonstration of MsrA activity in wild-type spores [13]. Here, we report an oxidative stress induced regulation pathway of *msrA *and *msrB *expression in *B. subtilis *and identify genes that are involved in the regulation of methionine oxidation and repair.

## Results

### Promoter localization of the *msrAB *operon

**Figure 1:**
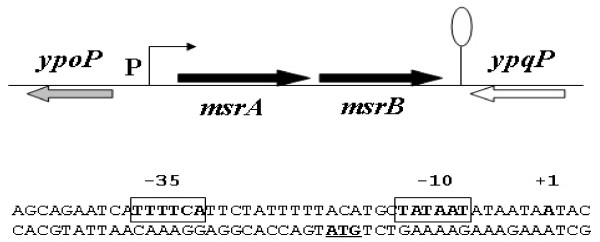
**Identification of the transcriptional start point of the *msrAB *operon**. The gene organization of *msrA *and *msrB *in *B. subtilis *chromosome is shown. The arrowheads indicate the direction of transcription. 'P' means promoter. The transcriptional start point (+1 site) is indicated in bold case. Predicted -10 and -35 regions are shown in bold and boxed. The translational start site is in bold case and underlined.

To identify the promoter of the putative *msrA*-*msrB *(formerly *yppQ*) operon, we mapped the transcriptional start sites of both *msrA *and *msrB *genes using the 5' RACE method. The same transcriptional start site was detected in each case. The *msrA *and *msrB *genes, which belonged to a common transcriptional unit, form therefore an operon in *B. subtilis*. The transcriptional start site of the *msrA*-*msrB *operon is located 30 nt upstream of the ATG translation start codon of *msrA *and regions similar to consensus -35 (TTTTCA) and -10 (TATAAT) boxes separated by 16 nt are found upstream of this start point (Fig. 1). In addition, the re-sequencing of the *msrAB *region confirmed that *msrA *and *msrB *are individual genes in *B. subtilis*. They are separated by a TAA stop codon (data not shown).

### The effect of oxidative stress on expression of the *msrAB *operon

The physiological role of MsrA and MsrB *in vivo *is protection against oxidative damage [14]. However, in most of the bacteria investigated so far, the expression of *msrA *has not been shown to be induced by oxidative stress [12]. To our knowledge, only two reports show an unambiguous regulatory effect of oxidative stress: the expression of *msrA *from *Xanthomonas campestris *pv. phaseoli [15] and Helicobacter pylori [16] is strongly induced by exposure to oxidants. To study the regulation of the *msrAB *operon in *B. subtilis *in response to oxidative stress, we used *lacZ *as a sensitive reporter of gene expression.

**Figure 2:**
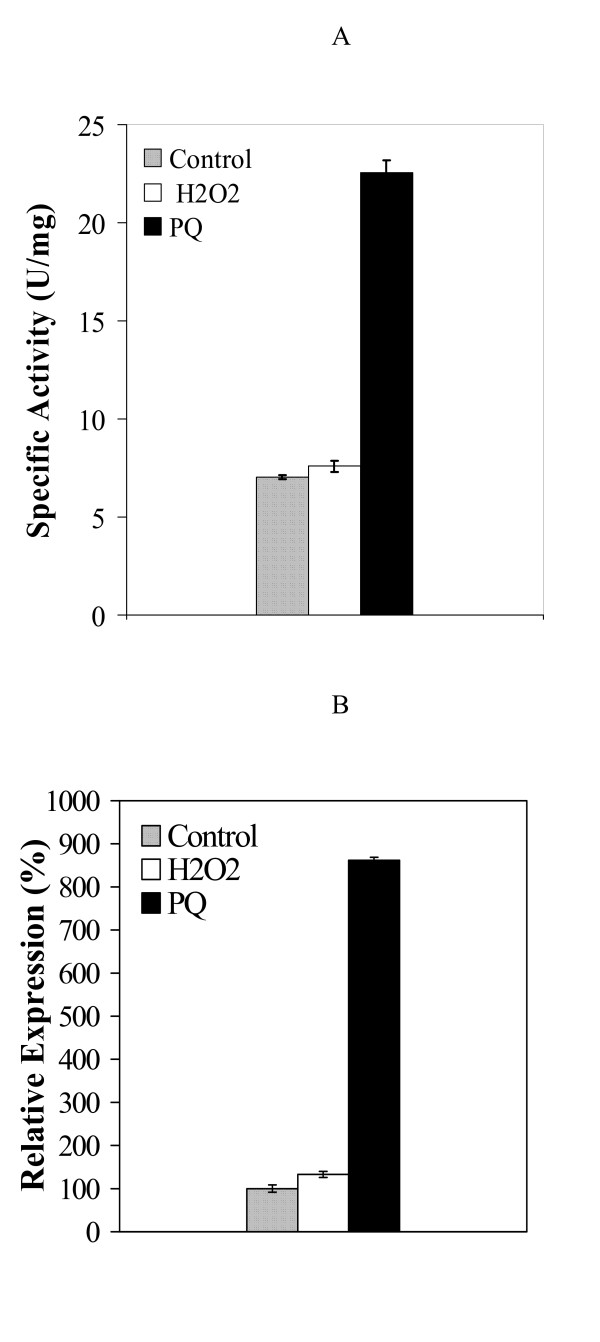
**Effect of oxidative stresses on the expression of *msrA***. A. β-galactosidase activity of the *msrA*::*lacZ *transcriptional fusion. BSY4546 (*msrA*::*lacZ*) was grown in ED minimal medium to mid-exponential phase and treated with or without PQ (100 μM) or H_2_O_2 _(100 μM) for 1.5 hours. B. Real time RT-PCR analysis of *msrA *expression in wild-type strain (168) subjected to oxidative stresses. 100% expression is defined as the expression of *msrA *without any oxidant treatment. Strain 168 was grown in ED minimal medium to mid-exponential phase and incubated with or without PQ (100 μM) or H_2_O_2_ (100 μM) for 15 min.

H_2_O_2 _had no effect on the expression of either a *msrA*::*lacZ *fusion or a *msrB*::*lacZ *fusion even at a concentration of 100 mM, which resulted in growth inhibition, while a 2-fold increase of a peroxide inducible *katA*::*lacZ *fusion expression was detected here in the presence of 200 μM H_2_O_2 _as previously reported [17]. In contrast, the addition of 100 μM PQ to the culture medium led to a 3.5-fold induction of the expression of the *msrA*::*lacZ *fusion as compared to the level observed in the absence of the oxidizing agent (Fig. 2A). Another superoxide generating molecule, diamide, had no effect (data not shown), prompting us to explore in some more depth the PQ effect, which was further confirmed by real time RT-PCR assays, with a 8-fold increase of the quantity of the *msrA *mRNA after PQ treatment (Fig. 2B). This induction factor is higher than that obtained with *lacZ *fusions, possibly because of the β-galactosidase sensitivity to PQ treatment. Thus, under the conditions tested in this study, the expression of the *msrAB *operon in *B. subtilis *was induced by PQ but not by H_2_O_2_, indicating the existence of a specific PQ-responsive regulation in *B. subtilis*.

### The global thiol stress response regulator Spx participates in PQ-specific induction of the *msrAB *operon

To identify regulators that may be responsible for the PQ-induced enhancement of *msrAB *expression, we studied the possible involvement of some previously reported oxidative stress response regulators in *B. subtilis*.

**Figure 3:**
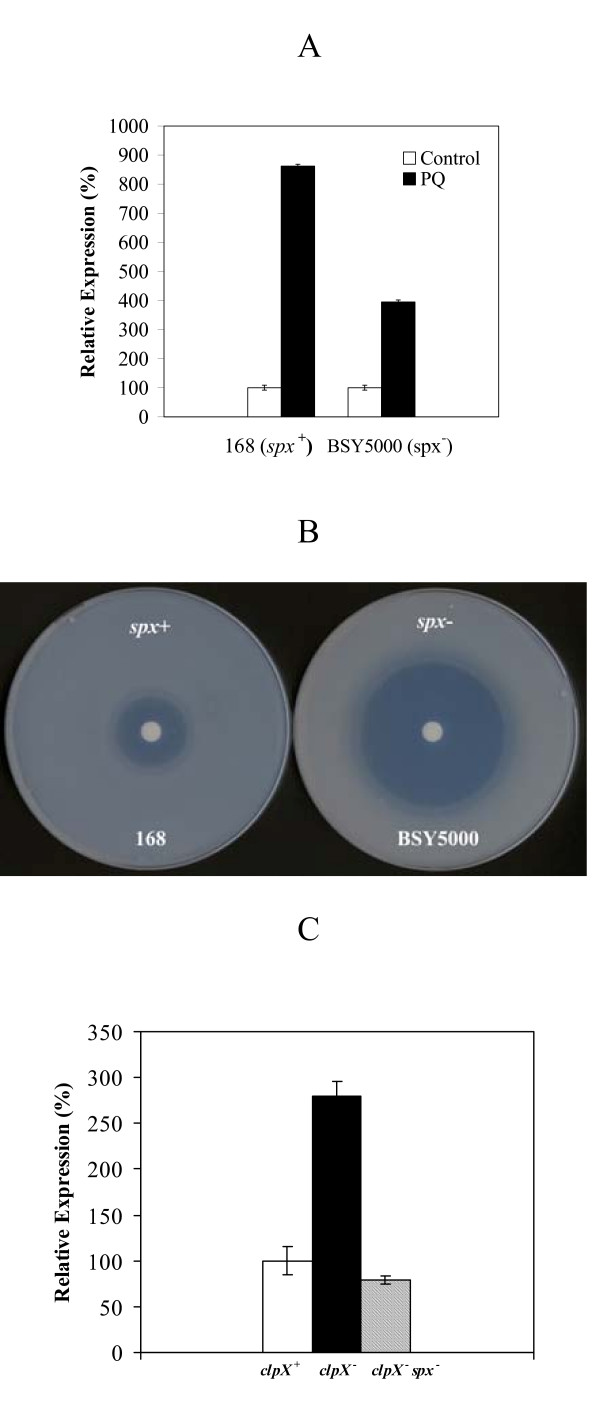
**Effect of Spx on PQ induced expression of *msrA***. A. RT-PCR analysis of the expression of *msrA *in wild-type strain (168) and *spx *mutant (BSY5000). 100% expression for each strain is defined as the expression of *msrA *without any oxidants treatment. Strains 168 and BSY5000 were grown in ED minimal medium to mid-exponential phase and incubated with or without PQ (100 μM) for 15 min. B. Disc inhibition assay of PQ induced oxidative stress for wild-type 168 strain and *spx *disrupted strain (BSY5000). C. *msrA *expression in a *clpX *mutant strain. RT-PCR analysis of the expression of *msrA *in wild-type 168 strain, *clpX *mutant (BSY6000) and *clpX*, *spx *double mutant (BSY4260). 100% expression is defined as the expression of *msrA *in wild-type strain 168. Strains were grown in LB rich medium to mid-exponential phase.

Spx is a global thiol stress response regulator in *B. subtilis*, and higher *msrA *expression has been observed in a transcriptome analysis when the Spx protein was maintained at a high concentration *in vivo *[18]. We tested the expression of the *msrA *gene in a *spx *mutant by real time RT-PCR assays. In the absence of oxidative stress, the *msrA *expression levels in strain BSY5000 (*spx*::*aphA-3*) and in the wild-type strain were similar (data not shown). This result was not surprising because Spx was barely detectable in the wild-type strain under these conditions [19]. However, in the *spx *mutant, the PQ-specific induction of *msrA *expression was reduced to approximately 50% of that observed in the *spx *wild-type strain (Fig. 3A) as shown by real time RT-PCR assays. Therefore, Spx was involved in a part of the PQ-specific induction of *msrA *expression. Interestingly, compared to the wild-type strain, the *spx *mutant is extremely sensitive to PQ (Fig. 3B), suggesting that Spx participates directly or indirectly in PQ response in *B. subtilis*. As reported previously, while present at a low level in the wild type, the Spx protein accumulates to a high concentration in a *B. subtilis clpX *mutant [20]. To substantiate the involvement of Spx in the regulation of the *msrAB *operon, we measured the expression of *msrA *in a *clpX *nullbackground or in a *clpX *and *spx *double mutant using real time RT-PCR (Fig. 3C). The quantity of the *msrA *transcript increased 3-fold in the *clpX *mutant as compared to that in the wild-type strain. However, the expression of the *msrA *transcript in the *clpX *and *spx *double mutant was similar to that in the wild-type strain. This indicates that Spx is required for the increased expression of *msrA *observed in the *clpX *mutant, supporting our finding that the Spx protein takes part in PQ induced *msrA *expression.

The increased expression of *msrA *observed in the *clpX *mutant suggested that the PQ challenge could lead to an increase of the Spx protein concentration *in vivo*. Indeed, our real time RT-PCR experiment showed a 6-fold increased expression of the *spx *gene after 100 μM PQ treatment (see Additional file 1). However, no significant change of the Spx amount in the cells was identified after PQ challenge when compared to the dramatic accumulation of Spx in a *clpX *mutant by western blotting analysis (data not shown). This suggests that some other mechanism than simply the quantity of the Spx protein is involved in the increase in *msrA *expression.

### The CXXC motif of Spx is essential for the activation of *msrAB *operon expression following PQ challenge

It has been proposed that the transcriptional activation effect of Spx on *trxA *and *trxB *expression could be due to the formation of an intra-molecular disulfide bond generated by oxidation of the two cysteine residues in the CXXC motif of the protein [21]. This prompted us to test the involvement of the Spx CXXC motif in the PQ-induced activation of the *msrAB *operon.

**Figure 4:**
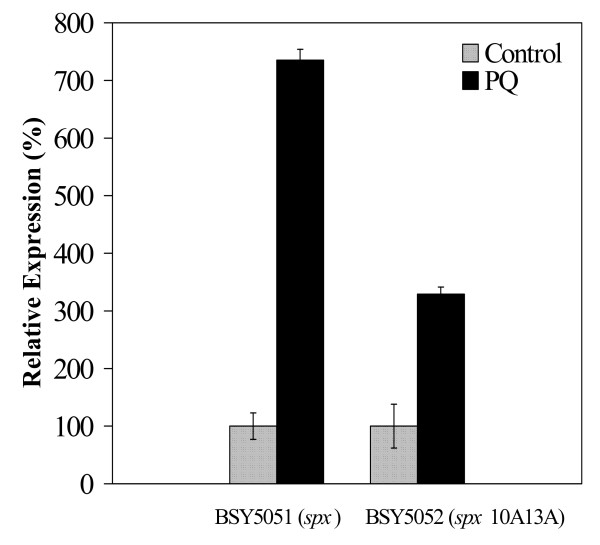
**Effect of modifications of the Spx CXXC motif on PQ induced expression of *msrA***. RT-PCR analysis of *msrA *expression in *spx *mutant complemented with wild-type *spx *(BSY5051) or *spx*10A13A allele (BSY5052) was performed. 100% expression for each strain is defined as the expression of *msrA *without any oxidant treatment. Strains were grown in ED minimal medium to mid-exponential phase and incubated with or without PQ (100 μM) for 15 min.

To this purpose, the *msrA *expression was tested by real time RT-PCR assays in a *spx *mutant that contained either the wild-type *spx *gene or the *spx *gene with a modified CXXC motif expressed under the control of a xylose inducible p*xyl *promoter (strains BSY5051 and BSY5052). After PQ treatment, the *msrA *expression was induced 7.5-fold in the presence of the wild-type *spx *gene (strain BSY5051) (Fig. 4). In contrast, the PQ induction was reduced to 3.5-fold in the presence of the *spx *gene containing an AXXA motif (strain BSY5052) (Fig. 4) as observed in the *spx *mutant (Fig. 3A). With PQ treatment, the expression of *msrA *in the presence of Spx containing an AXXA motif behaved like that of the *spx *null strain, indicating that the CXXC motif of Spx was required for the PQ-specific induction of the *msrAB *operon.

### The CXXC motif of Spx was modified after PQ treatment

In many cases, CXXC motifs in proteins are highly sensitive to oxidants and can work as regulatory switches [22]. Therefore, this motif in Spx might be modified after PQ treatment to work as a regulatory switch.

To test this hypothesis, we monitored the status of the Spx protein following PQ challenge. To this purpose, we constructed strain BSY2534, which contains a translational fusion between *spx *and a HA-tag codons expressed under the control of a xylose inducible p*xyl *promoter. The Spx-HA fusion protein was detected by western blotting analysis with an antibody against the HA-tag. Similar analysis was performed using strains producing Spx-HA proteins modified in the CXXC motif: strain BSY2531 (Spx10A-HA), strain BSY2533 (Spx13A-HA) and strain BSY2535 (Spx10A13A-HA). To show that the HA tag did not interfere with the function of Spx, we verified that the presence of the HA-tag did not modify the Spx-dependent regulation of *msrA *expression during PQ stress (see Additional file 2).

**Figure 5:**
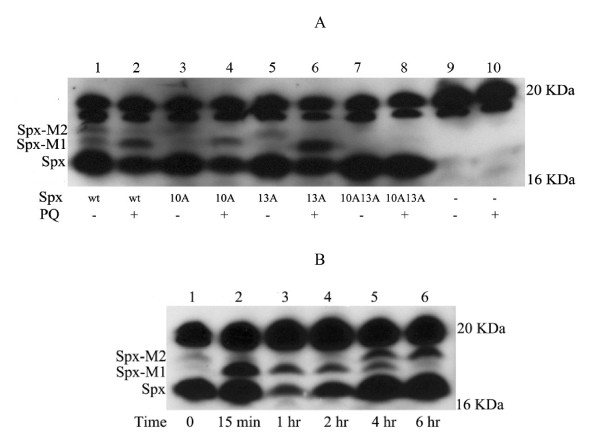
**Analysis and characterization of PQ modification effect on Spx and PQ modified form of Spx**. A. PQ-induced modifications of the CXXC motif of Spx. Strains producing wild-type and CXXC motif mutated Spx-HA fusion proteins were treated with or without 100 μM PQ for 15 min. Crude cell extracts from each strain were analyzed by western blot using an antibody against HA. lane 1–2 (wt): strain BSY2534 producing Spx-HA protein; lane 3–4 (10A): strain BSY2531 producing Spx10A-HA protein; lane 5–6 (13A): strain BSY2533 producing Spx13A-HA protein; lane 7–8 (10A13A): strain BSY2535 producing Spx10A13A-HA protein; lane 9–10: vector control (strain BSY2530 that did not express any HA fusion protein). lane 2, 4, 6, 8, 10: with PQ treatment; lane 1, 3, 5, 7, 9: without PQ treatment. The modified forms of Spx were marked as Spx-M1, Spx-M2 and native forms of Spx were marked as Spx. B. Time course changes of PQ-induced modifications of Spx *in vivo*. Strain BSY2534 producing Spx-HA was incubated with 100 μM PQ for different time (0–6 hr). 40 μg protein from cell crude extracts obtained at each time point was analyzed by western blot using anti-HA antibody. lane 1: 0 min; lane 2: 15 min; lane 3: 1 hr; lane 4: 2 hr; lane 5:4 hr; lane 6: 6 hr.

In the western blot (Fig. 5A), two strong signal bands obtained in the 20 KDa region in each lane were non-specific bands (lane 1–10), and three forms of Spx-HA proteins (lanes 1 and 2) were detected compared to the negative control without a Spx-HA tagged protein (lanes 9 and 10). The major band corresponded to the predicted molecular mass of Spx-HA (around 17 KDa) while two additional forms named Spx-M1 and Spx-M2 appeared in the higher molecular mass region (lane 1). Interestingly, after PQ treatment (lane 2), the amount of Spx-M1 significantly increased but the amount of the native Spx and to a less extend of the Spx-M2 decreased as compared to the untreated sample (lane 1). Similar patterns were observed with Spx10A (lanes 3 and 4) and Spx13A (lanes 5 and 6) proteins. In both cases, PQ treatment led to an increase of the amount of Spx10A-M1 and Spx13A-M1 forms. In contrast, no Spx-M1 or Spx-M2 forms were detected for the Spx10A13A protein with and without PQ treatment (lanes 7 and 8). These results strongly suggested that PQ treatment led to a post-translational modification of Spx involving the cysteine rich motif (CXXC) and that at least one cysteine residue of the CXXC motif was required for the PQ mediated modification of Spx. We then studied the time course of changes in Spx *in vivo *after PQ treatment. As shown in Fig. 5B, a 15 minute treatment led to the highest amount of Spx-M1 (lane 2) *in vivo*. The amount of Spx-M1 further decreased until it totally disappeared at six hours after treatment while the Spx-M2 accumulated between four and six hours (lane 6). In parallel, the amount of the native form of Spx decreased to reach the lowest level one hour after treatment (lane 3), then started to increase (lane 4–6). At six hours, it reached a level similar to that detected before PQ treatment (Lane 1). Therefore, both the modified form of Spx and the native Spx showed dynamic changes *in vivo *after a PQ treatment.

In many cases, oxidative reagents modify the CXXC motifs of proteins through the formation of disulfide bonds [23]. This modification is reversible *in vitro *after incubation with reducing reagents, such as DTT [24]. Therefore, we tested whether PQ induced modification of the CXXC motif of Spx could be reversed by DTT. The total cellular extracts of strain BSY2534 obtained after a PQ treatment were incubated with or without 50 mM DTT for different time. Interestingly, the Spx-M1 form of Spx did not disappear after DTT treatment under the conditions tested (data not shown) indicating that this modification is unlikely to be mediated by disulfide bonds formation.

## Discussion

Factors involved in the regulation of *msrA *and *msrB *genes in bacteria are still largely unknown [12]. In the present study, we demonstrated that the expression of the *msrAB *operon in *B. subtilis *was significantly induced under PQ-mediated oxidative stress conditions (Fig. 2). PQ is an oxidant that is commonly recognized to generate superoxide *in vivo*. It may also generate other ROS, including singlet oxygen. However, in our experiments a *msrAB *mutant was not more sensitive to PQ, H_2_O_2_, or diamide treatment than the wild-type strain (data not shown), suggesting that methionine sulfoxide reductases in *B. subtilis *are not the key enzymesagainst oxidative stresses. Remarkably, in our hands, *msrAB *expression was not regulated by H_2_O_2 _oxidative stress (Fig. 2). The finding that the response to PQ and H_2_O_2 _induced oxidative stresses differ is in line with some previous observations in different bacterial species. For example, the oxidant-induced expression of *msrA *in *X. campestris *varies according to the nature of the oxidants used [15]. Furthermore, in *B. subtilis *many genes were reported to selectively respond either to PQ or to H_2_O_2 _[25,26]. Similar to the situation of the *msrAB *operon, the expression of *cysK *and other genes involved in cysteine metabolism is only induced by PQ but not by H_2_O_2 _[25,26]. PerR, the specific peroxide responsive regulator in *B. subtilis*, is not involved in the regulation of the *msrAB *operon, in agreement with previous results indicating that *msrAB *does not belong to the PerR regulon [27]. In addition, a peroxide induced sigma B-dependent regulation of *msrAB *seems unlikely since no sigma B promoter is found upstream of the *msrAB *operon ([28,29], this work).

In the present study, we showed that Spx, a global transcriptional regulator of the disulfide specific oxidative stress response in *B. subtilis *plays a central role in the PQ-specific induction of *msrAB *expression. Surprisingly, diamide, a reagent that causes disulfide stress in organisms, did not induce the expression of *msrAB *in all the conditions tested, while we found a complex involvement of Spx, originally identified as a disulfide stress regulator (data not shown). This finding seems to be in contradiction with the fact of the participation of Spx in the PQ induced expression of *msrAB*. Several reasons might account for that observation: (i) disulfide bond formation might not involve in this PQ induced expression of *msrAB *(see below); (ii) Spx could respond to other oxidative stress beside disulfide stress; (iii) the expression of *spx *is regulated in a particularly complex way, with effectors that might compensate each other action on *msrAB *expression [30]. As a case in point, the *spx *mutant showed more sensitivity to PQ treatment compared to the wild-type stain (Fig. 3B).

In line with (iii), the Spx mediated regulatory effect on *msrAB *is only partial since PQ has a residual induction effect on *msrA *in a *spx *mutant (Fig. 3A). Therefore, besides Spx, there were some unidentified PQ responding factors regulating *msrAB *expression, or PQ could affect *msrAB *mRNA stability *in vivo*. More studies are required to unravel the processes underlying these complex effects.

Further experiments indicated that the PQ effect on Spx occurs at the post-translational level and that the CXXC motif of Spx may be a target of PQ oxidation because this motif was essential for the PQ-induced enhancement of the *msrA *expression mediated by Spx (Fig. 4). This hinted the possibility that PQ activates Spx through an oxidative modification of its CXXC motif.

Interestingly, PQ challenge brought a significantly increased amount of a new form of Spx, Spx-M1. This form, which has an apparent mass 400 Da higher than that of the native Spx, probably corresponds to a post-translational modification of Spx (Fig. 5A). The Spx-M1 form is still observed with Spx10A or Spx13A but disappears in a strain producing the Spx10A13A protein. This indicates the requirement for at least one of the two cysteines of the CXXC motif to observe the PQ-induced modification of Spx. So far, we failed to purify sufficient amounts of Spx-M1 in *B. subtilis *to identify the nature of this modification. Since the amount of Spx-M2 only showed a moderate change in all the conditions tested here, extensive studies on Spx-M2 will be carried out in a further work.

In *Xanthomonas campestris*, a cysteine motif of the OhrR regulator that senses hydroperoxides is modified as cysteine-sulfenic acid which rapidly reacts with another cysteine of the protein [31]. The OhrR regulator of *B. subtilis *has recently been shown to be modified in the same way after oxidative stress with the formation of a disulfide bond (S-thiolation) between the Cys15 of OhrR and a new but still uncharacterized low molecular mass motif of 398 Da [27]. This chemical modification has also been detected in *Bacillus anthracis *[32]. In the present report the size increase of Spx after PQ treatment could correspond to the same sulfenic acid modification followed by reaction with a thiol present in the cytoplasm of *B. subtilis*, playing the role of glutathione in other bacteria. However, since the PQ modified form of Spx is resistant to DTT under the conditions tested (data not shown), a modification by S-thiolation seems unlikely. We cannot exclude that a derivative of PQ might have reacted with an activated thiol of Spx. Finally, we could not completely rule out the possibility that conformational changes brought by PQ could be responsible for the different forms of Spx detected. In any event, the direct covalent modification or conformational modification of Spx seems to be removed *in vivo*, and the amount of native Spx also showed a dynamic change after PQ treatment (Fig. 5B). In more details, with short PQ treatment time, the native Spx showed no much changes (lane 2), but longer PQ treatment seemed to trigger the degradation of the native Spx *in vivo *(lane 3), which fits observations reported previously on the effects of oxidative stress on proteins *in vivo*, which is causing protein degradation [33,34]. It appears that subsequent to a degradative process Spx was regenerated *in vivo *after the cells have adapted to the PQ treatment (lane 4–6). These facts suggested that Spx protein experienced complex processes of modification, degradation and regeneration *in vivo *following PQ treatment.

## Conclusion

In summary, this study reports a Spx mediated PQ-specific regulation pathway of the *msrAB *operon in *B. subtilis*. PQ induces the expression of *msrAB *partially through its possible oxidation of the Spx CXXC motif. More studies are expected to discover the molecular mechanism of PQ-induced modification of Spx as well as other factors involved in the PQ-specific induction of the *msrAB *operon.

## Methods

### Bacterial strains, plasmids, and culture conditions

*E. coli *and *B. subtilis *strains and plasmids used in this work are listed in Table 1. *E. coli *cells were grown in LB medium. *B. subtilis *cells were grown in LB medium, sporulation (SP) medium (0.8% nutrient broth, 1 mM MgSO_4_, 13.4 mM KCl, 0.5 mM CaCl_2_, 10 μM MnCl_2_, 13.4 μM ferric ammonium citrate), or ED minimal medium (6 mM K_2_HPO_4_, 4.4 mM KH_2_PO_4_, 27 mM glucose (or fructose), 0.3 mM Na_3_-citrate, 15 mM L-glutamine, 0.244 mM L-tryptophan, 33.5 μM ferric ammonium citrate, 1 mM MgSO_4_, 4 mM MgCl_2_, 0.25 mM CaCl_2_, 10 μM MnCl_2_). Antibiotics were added with the following concentration when required: ampicillin (Amp), 100 μg ml^-1 ^for *E. coli*; spectinomycin (Spc), 60 μg ml^-1 ^for both *E. coli *and *B. subtilis*; erythromycin (Ery), 1 μg ml^-1^, chloramphenicol (Cam), 5 μg ml^-1^, and kanamycin (Kan), 5 μg ml^-1 ^for *B. subtilis*. When xylose was added (at 0.5% (w/v) final concentration), fructose was used as carbon source. Solid media were prepared by adding agar to 1.5% (w/v) to the respective liquid media. Amylase activity was detected after growth of *B. subtilis *strains on Tryptose Blood Agar Base (TBAB, Difco) supplemented with 10 g/liter hydrolyzed starch (Sigma). Starch degradation was detected by sublimating iodine onto the plates. All experiments were performed in accordance with the European regulation requirements concerning the contained use of Genetically Modified Organisms of Group-I (French agreement N°2735).

**Table 1:** Strains and plasmids used in this study

**Strains**	**Genotype**	**Source or Reference**
*Escherichia coli*		

DH5α	*F-, Δ80dlacZΔM15, Δ(lacZYA-argF)U169, deoR, recA1, endA1*	Laboratory collection
	*hsdR17(rk-, mk+), phoA, supE44, λ -, thi-1, gyrA96, relA1*	

*Bacillus subtilis*		

168	*trpC*2	Laboratory collection
BFS2842	*trpC*2 *spx*::*lacZ erm*	Functional analysis project^a^
BSY2530	*trpC*2 *amyE*:: pX vector *cat*	This work
BSY2531	*trpC*2 *amyE*:: (p*xyl*-*spx*10A-HA) *cat*	This work
BSY2533	*trpC*2 *amyE*:: (p*xyl*-*spx*13A-HA) *cat*	This work
BSY2534	*trpC*2 *amyE*:: (p*xyl*-*spx-*HA) *cat*	This work
BSY2535	*trpC*2 *amyE*:: (p*xyl*-*spx*10A13A-HA) *cat*	This work
BSY4260	*trpC*2 *clpX*::*aphA*-3 *spx*::*lacZ erm*	This work
BSY4546	*trpC*2 *msrA*::*lacZ cat*	This work
BSY5000	*trpC*2 *spx*::*aphA*-3	This work
BSY5051	*trpC*2 *spx*::*aphA*-3 *thrC*::(p*xyl*-*spx*) *spc*	This work
BSY5052	*trpC*2 *spx*::*aphA*-3 *thrC*::(p*xyl*-*spx*10A13A) *spc*	This work
BSY5531	*trpC*2 *spx*::*aphA*-3 *amyE*:: (p*xyl*-*spx*10A-HA) *cat*	This work
BSY5533	*trpC*2 *spx*::*aphA*-3 *amyE*:: (p*xyl*-*spx*13A-HA) *cat*	This work
BSY5534	*trpC*2 *spx*::*aphA*-3 *amyE*:: (p*xyl*-*spx-*HA) *cat*	This work
BSY5535	*trpC*2 *spx*::*aphA*-3 *amyE*:: (p*xyl*-*spx*10A13A-HA) *cat*	This work
BSY6000	*trpC*2 *clpX*::*aphA*-3	This work

**Plasmids**	**Description**	**Source or Reference**

pDIA5307	*lacZ *transcriptional fusion vector, Amp^R ^Cam^R^	[37]
pDG784	*aphA-*3 gene delivery vector, Kan^R^	[38]
pXT	gene expression vector Amp^R^Spc^R^	[40]
pX	gene expression vector Amp^R^Cam^R^	[41]
pCHY109	pXT *spx *Amp^R^Spc^R^	This work
pCHY110	pXT *spx*10A13A Amp^R^Spc^R^	This work
pCHY111	pX *spx*10A-HA Amp^R^Cam^R^	This work
pCHY113	pX *spx*13A-HA Amp^R^Cam^R^	This work
pCHY115	pX *spx*10A13A-HA Amp^R^Cam^R^	This work
pCHY253	pX *spx*-HA Amp^R^Cam^R^	This work
pCHY4546	pDIA5307 *msrA*::*lacZ *Amp^R ^Cam^R^	This work

^a^This strain has been constructed during the frame of the project for the functional characterization of the genome of *B. subtilis *in Japan [46].

*aphA*-3 is a kanamycin resistance gene; *cat *is a chloramphenical acetyl transferase gene; *spc *is a spectinomycin resistance gene, *erm *is a erythromycin resistance gene.

### DNA techniques

DNA purification, restriction enzyme digestion, ligation and transformation of *E. coli *were performed according to standard protocols [35]. For cloning purpose, *YieldAce *DNA polymerase (Stratagene) was used. All the primers used for PCR in this study are listed in Table 2. DNA sequence of plasmid constructed in this work was determined using the dideoxy-chain termination method and Thermo Sequenase Kit (Amersham Pharmacia Biotech). *B. subtilis *cells were transformed with either plasmid DNA or chromosomal DNA following the two-step protocol as described previously [36].

**Table 2:** Primers used in this study

**Primer Name**	**Sequence (5'-3')**
CHY13	GATAAACCCAGCGAACCATTTGAGGTG
CHY14	GATACAAATTCCTCGTAGGCGCTCGGGAC
CHY45	CCGGAATTCGAAATCGCCACATTTGCAGG
CHY46	CGCGGATCCTTATTTAGCCCCCCAATGCTCG
CHY101	AAGCCCATATTGCTCGAGGTGG
CHY102	TATCACCTCAAATGGTTCGCTGGGTTTATCCAGCTTGGTGATGTGTATAGTG
CHY103	GGTCCCGAGCGCCTACGAGGAATTTGTATCAGAAGCACAGCGTTTGGCAAAC
CHY104	GGAACATTTATTGCCGTTGCC
CHY109	AAGGAGGAAGCAGGTATGGTTACACTATACACATC
CHY110	GACACGCACGAGGTTTAGTTTGCCAAACGCTGTG
CHY111	CTCGCCTTTCTGCATGAAGTCGCGCTTGGTGATGTGTATAGTG
CHY112	CACCAAGCGCGACTTCATGCAGAAAGGCGAG
CHY113	GAGCCACGCTCTCGCCTTTCTCGCTGAAGTACAGCTTGGTGATG
CHY114	ACTTCAGCGAGAAAGGCGAGAGCGTGGCTC
CHY115	GAGCCACGCTCTCGCCTTTCTCGCTGAAGTCGCGCTTGGTGATGTGTATAGTG
CHY128	GTCTGCAAATGCAAGGCATG
CHY129	TATCACCTCAAATGGTTCGCTGGGTTTATCCAGCTACAAGCTTACGAACCTG
CHY130	GGTCCCGAGCGCCTACGAGGAATTTGTATCGCAACTGTGACACACGGAGAG
CHY131	AGTTCCACAAAGACAGCCTG
CHY134	CGGCCATACTGAAAACCCTA
CHY135	ATCTGTCGGATCGATTTGCT
CHY137	GCAGTAACGAAGTCCGTTTG
CHY140	CCGCATGGTTCAAACATAAA
CHY141	CGTCAGACTTTCGTCCATTG
CHY173	CCACTTCTTCTTCAATCGGC
CHY253	GGACTAGTAGAGGAGTGAAGATGAATGG
CHY254	CGCGGATCCTTAAGCGTAATCCGGAACATCGTATGGGTAGTTTGCCAAACGCTGTGCTTC
YGSP3	TTTCCGCCAGACGTTGCTTG
YGSP4	GCATCTGTCGGATCGATTTG

### Plasmids and strains constructions

To construct *msrA *transcriptional fusions with the *lacZ *gene, DNA fragments of the *msrA *gene (nucleotides from + 16 to +534 relative to the translation start point) were amplified by PCR from genomic DNA of *B. subtilis *168 using primers CHY45 and CHY46, then inserted into the *Eco*RI and *Bam*HI sites of plasmid pDIA5307 (*in situ lacZ *transcriptional fusion vector) [37], producing plasmid pCHY4546. The plasmid pCHY4546 was introduced into the chromosome of *B. subtilis *168 by a single crossover event, giving strain BSY4546. Strain BSY4546 was further verified by PCR. In strain BSY4546, the *lacZ *gene was inserted after the translation stop codon of *msrA*, therefore a complete copy of *msrA *is present.

The *spx *and *clpX *genes were individually disrupted by a kanamycin resistance cassette using three overlapping fragments with primers running PCR. DNA fragments I and II, which correspond to the upstream (nucleotides -519 to +29 relative to *spx *translation start point) and downstream (nucleotides -25 to +565 relative to *spx *translation stop point) regions of *spx*, were amplified by PCR from genomic DNA of *B. subtilis *168 using primers CHY101 and CHY102, or CHY103 and CHY104, respectively. DNA fragments I and II, which correspond to the upstream region (nucleotides -580 to +85 relative to *clpX *translation start point) and the downstream region (nucleotides -81 to +543 relative to *clpX *translation stop point) of *clpX*, were amplified by PCR from genomic DNA of *B. subtilis *168 using primers CHY128 and CHY129, or CHY130 and CHY131, respectively. DNA fragment III (kanamycin cassette gene (*aphA*-3) (1486 bp)) for both of them was amplified by PCR from plasmid pDG784 [38] using primers CHY13 and CHY14. In each case, the three purified PCR fragments were mixed in equal amounts. Primers CHY101 and CHY104 were used to carry out the three fragments PCR for *spx*, while primers CHY128 and CHY131 were used for *clpX*. The resulting PCR fragments (2624 bp for *spx *and 2775 bp for *clpX*) were purified and used individually to transform *B. subtilis *168. Kanamycin resistant (Kan^R^) transformants selected on SP plates containing kanamycin were further verified by PCR, resulting in the *spx *and *clpX *knockout strains BSY5000 and BSY6000. The chromosomal DNA of strains BSY5000 and BSY6000 was prepared and used to transform strains BSY4546 respectively, resulting in strains BSY4550 and BSY4560.

To obtain a *spx *and *clpX *double mutant strain, the chromosomal DNA of the *clpX *knockout strain BSY6000 was transformed into a *spx *null mutant strain BFS2842 (constructed in the functional analysis project of the *B. subtilis *genome [39]). Consequently, kanamycin and erythromycin resistant colonies were selected, resulting in the *spx *and *clpX *double mutant strain BSY4260.

To obtain a *spx *gene containing two mutations modifying the CXXC motif of Spx by AXXA (C10A, C13A), the upstream part of the *spx *coding sequence (nucleotides -519 to +60 relative to the translational start site) and the downstream part of *spx *(nucleotides +30 to +961 relative to the translational start site) were amplified by PCR from genomic DNA of *B. subtilis *168 using primers CHY101 and CHY114, or CHY115 and CHY104, respectively, introducing the point mutations C10A and C13A. These two purified PCR fragments were mixed in equal amounts, and primers CHY109 and CHY110 were used to carry out overlap PCR. The resulting PCR fragment contained the complete coding sequences of *spx *(nucleotides -36 to +467 relative to the translational start site) containing mutations C10A and C13A. The complete coding sequence of *spx *(nucleotides -36 to +467 relative to the translational start site) was also amplified by PCR from genomic DNA of *B. subtilis *168 using primers CHY109 and CHY110. Plasmid pXT [40] was then used to clone the wild-type and the modified *spx*10A13A genes under the control of the xylose-inducible promoter. The PCR fragments were inserted between the *Eco*RI and *Bam*HI sites of plasmid pXT, resulting in plasmid pCHY109 and PCH110. Plasmids pCHY109 and pCHY110 were digested by *Sca*I and used to transform strains BSY 5000. The p*xyl*-*spx *gene or the p*xyl*-*spx*(10A,13A) gene was inserted by a double crossover event at the *thrC *locus of these strains, giving rise to strains BSY5051 and BSY5052.

To construct a C-terminal HA (Hemagglutinin, YPYDVPDYA)-tag fusion with the Spx protein, a DNA fragment corresponding to the *spx *gene (nucleotides from -16 to +393 relative to the translation start point) was amplified by PCR from genomic DNA of *B. subtilis *168 using primers CHY253 and CHY254, leading to a translational fusion between *spx *and HA tag codons at the N-terminal. Plasmid pX [41] was used to clone the *spx *gene fused with HA codons under the control of a xylose-inducible promoter. This PCR fragment was then inserted into the *Spe*I and *Bam*HI sites of plasmid pX, producing plasmid pCHY253. This plasmid was digested by *Sca*I and integrated into the *amyE *locus on the chromosome of *B. subtilis *168 by a double crossover event, giving strain BSY2534. The loss of amylase activity was detected as reported before [42]. Strain BSY2534 was further verified by PCR. Chromosomal DNA of the BSY2534 strain was transformed into the *spx *mutant strain BSY5000, giving rise to strain BSY5534.

To obtain a *spx *gene containing three mutations modifying the CXXC motif of Spx-HA fusion protein by AXXC (C10A), CXXA (C13A) and AXXA (C10AC13A), the upstream part of the *spx *coding sequence(nucleotides -519 to +60 relative to the translational start site) were amplified by PCR from genomic DNA of *B. subtilis *168 using primers CHY101 and CHY111, CHY101 and CHY113, and CHY101 and CHY114, individually, and the individually corresponding downstream part of *spx *(nucleotides +30 to +393 relative to the translational start site) were amplified by PCR from genomic DNA of *B. subtilis *168 using primers CHY112 and CHY254, CHY114 and CHY254, and CHY115 and CHY254, introducing the point mutations C10A, C13A, and C10AC13A. Each of above two corresponding purified PCR fragments was mixed in equal amount, and primers CHY253 and CHY254 were used to carry out overlap PCR. The resulting PCR fragments contained the complete coding sequences of *spx *with the HA tag gene containing mutations at C10A, C13A and C10AC13A, individually. These PCR fragments were inserted between the *Spe*I and *Bam*HI sites of plasmid pX, resulting in plasmids pCHY111, pCH113 and pCHY115. These plasmids were digested by *Sca*I and used to transform strains 168 and BSY 5000, respectively. The *spx *with HA tag gene, *spx*10A with HA tag gene, *spx*13A with HA tag gene and the *spx*10A13A with HA tag gene was inserted by a double crossover event at the *amyE *locus of these strains, giving rise to strains BSY2531, BSY2533, BSY2535 and BSY5531, BSY5533, BSY5535. To make a control, the plasmid pX was digested by *Sca*I and inserted into the *amyE *locus of strain 168 by a double crossover event, producing strain BSY2530.

### Oxidative stress induction, RNA extraction and β-galactosidase assays

Overnight *B. subtilis *cultures in LB were diluted to an OD 600 nm of 0.1 in ED minimal medium and cultured at 37°C. When OD 600 nm reached 0.6, cell cultures were split into three aliquots with one incubated with H_2_O_2 _(100 μM), one incubated with PQ (100 μM), and one kept as a control. In some cases, cell cultures were split into two aliquots, treated with or without PQ (100 μM), respectively. After 15 min treatment, 10 ml of cells were centrifuged at 7000 rpm at 4°C for 1 min and RNA was extracted as reported previously [43].

β-galactosidase assay was performed as described by Miller [44]. One Unit of β-galactosidase was defined as the amount of enzyme that produced 1 nmol of *o*-nitrophenol per min at 28°C. Specific activity was expressed in Units per mg protein. Protein concentration was determined by the Bradford's method [45]. The experiments were performed in triplicates.

### Real time PCR assays and 5' RACE analysis

3 μg of the DNase I-treated total RNA extracted from different strains treated with or without oxidants was reverse-transcribed to generate cDNA using AMV Reverse Transcriptase (Roche) and random primer pDN(6) (Roche) according to the manufacturer's instructions. Real-time PCR (RT-PCR) analyzing the transcription of *msrA *and 16S rRNA using each cDNA as a template was performed using SYBR Green PCR Master Mix and SDS 2.1 software on a PRISM 7900 HT platform (Applied Biosystems). Relative quantification of *msrA *transcription in various conditions and various strains was analyzed by the comparative Ct (threshold cycle) method according to the manufacturer's instructions. The internal control used here was 16S rRNA. Primers used for *msrA *and 16S rRNA are CHY134 and CHY135, and CHY140 and CHY141, respectively.

The start site of the *msrA *or *msrB *transcript was identified by 5' RACE System for Rapid Amplification of cDNA Ends kit (Invitrogen). The first strand cDNA was generated with primers YGSP3 (complementary to nucleotides +345 to +364 relative to *msrA *translation start point) and CHY137 (complementary to nucleotides +325 to +345 relative to *msrB *translation start point) using 2 μg total RNA from *B. subtilis *strain 168 as template. After dC-tail treatment of the two cDNA obtained, the *msrA *cDNA was further amplified by PCR using the Abridged Anchor Primer from the kit and the nested primer YGSP4 (nucleotides +239 to +257 relative to *msrA *translation start point) while the *msrB *cDNA was amplified using the Abridged Anchor Primer from the kit and the nested primer CHY173 (nucleotides +206 to +226 relative to *msrB *translation start point). The two resulting PCR fragments were loaded on 1% agarose gel, and a single band was detected in each case. The two PCR fragments were sequenced to determine the transcriptional start site.

### Western blotting analysis

To study the *in vivo *effect of PQ-induction on the Spx protein in *B. subtilis*, strains BSY2534, BSY2531, BSY2533, BSY2535, BSY2530, which contain Spx-HA, Spx (10A)-HA, Spx (13A)-HA, Spx (10A13A)-HA fusion proteins and the negative vector control, individually, were grown in ED minimal medium to an OD 600 nm of 0.6. Then, each strain culture was divided into two fractions. One was incubated with 100 μM PQ for 15 minutes, and the second was kept as a control. 50 ml of cells from each culture was harvested by centrifugation, resuspended in 1 ml of 10 mM TrisHCl, pH 6.8 with 40 μl of Complete Protease Inhibitor (Roche), and disrupted by sonication. The cell debris was removed by centrifugation at 16,000 g for 40 min. Protein concentration was determined by the Bradford's method [45]. 40 μg of protein crude extracts from each culture was loaded onto a 17% SDS-PAGE. Proteins were subsequently transferred to nitrocellulose by iBlot™ Dry Blotting System (Invitrogen) and incubated with Anti-HA-Peroxidase antibody (Roche). Blots were developed with Amersham ECL Plus Western Blotting Detection System (GE healthcare) and signals were detected by a phosphorimager.

To study the effect of the addition of the dithiothreitol (DTT) on the PQ-induced modifications of Spx, the above crude extracts of strain BSY2534 obtained after 15 min PQ (100 μM) treatment was incubated with or without 50 mM DTT at 37°C for 0 hr, 2 hr or 4 hr. Then 40 μg protein obtained at each time point was loaded onto a 17% SDS-PAGE. A western blotting experiment was performed and Spx-HA was detected with Anti-HA-Peroxidase antibody as described above.

To monitor the dynamic changes of the different Spx forms *in vivo*, strain BSY2534 was grown in ED minimal medium to an OD 600 nm of 0.6, and was treated by 100 μM PQ. At different time point (from 0 to 6 hours), 50 ml cell culture was collected and disrupted. 40 μg protein from each cell crude extracts was analyzed by western blot using the anti-HA-Peroxidase antibody as described above.

### Disc inhibition assay

*B. subtilis *strains wild-type 168 and *spx *disrupted strain BSY5000 were grown in ED minimal medium. 2 ml of cell culture from each strain at mid-logarithmic phase was poured evenly onto ED minimal medium plate. After 5 min, the liquid cell culture on the plate surface was removed carefully. When the plate was dried, a filter disc (diameter: 0.7 cm) was placed in the middle of the plate and impregnated with 8 μl of 10 mM PQ. The diameters of the inhibition zone were measured after the plates were incubated at 37°C for 16 hours.

## Authors' contributions

AD and CHY designed the whole experiments. AS, OF, IM–V and YPW helped to design some of the experiments. CHY carried out all the experiments. CHY and AD draft this manuscript and all the others contributed in the writing. All authors approved the final manuscript.

## Supplementary Material

Additional file 1 **Figure S1 – Effect of PQ on the expression of *spx***. RT-PCR analysis of *spx *expression in wild-type strain (168) subjected to a PQ induced oxidative stress. 100% expression is defined as the expression of *spx *without PQ treatment. Strain 168 was grown in ED minimal medium to a mid-exponential phase and incubated with or without PQ (100 μM) for 15 min. Primers CHY255 (5'tgcaagatttgtaccgcttg3') and CHY256 (5'tgcaagatttgtaccgcttg3') specific for *spx *gene were used. Click here for file

Additional file 2 **Figure S2 – Wild-type and CXXC motif mutated Spx-HA fusion proteins effect on PQ induced expression of *msrA***. Real time RT-PCR analysis of *msrA *expression in a *spx *mutant (BSY5000) and in *spx *mutant complemented with either *spx-*HA (BSY5534), or *spx*10A-HA (BSY5531), or *spx*13A-HA (BSY5533), or *spx*10A13A-HA (BSY5535) was performed. Each strain was grown in ED minimal medium to a mid-exponential phase and treated with or without 100 μM PQ for 15 min. 100% expression for each strain is defined as the expression of *msrA *without any oxidants treatment. Click here for file
