# Supplementary material for: Spx mediates oxidative stress regulation of the methionine sulfoxide reductases operon in Bacillus subtilis
Source: BMC Microbiol. 2008 Jul 28;8:128. doi: 10.1186/1471-2180-8-128 (PMC2518928; doi:10.1186/1471-2180-8-128)
Supplement: Supplementary file 2 [file 1471-2180-8-128-S2.doc]

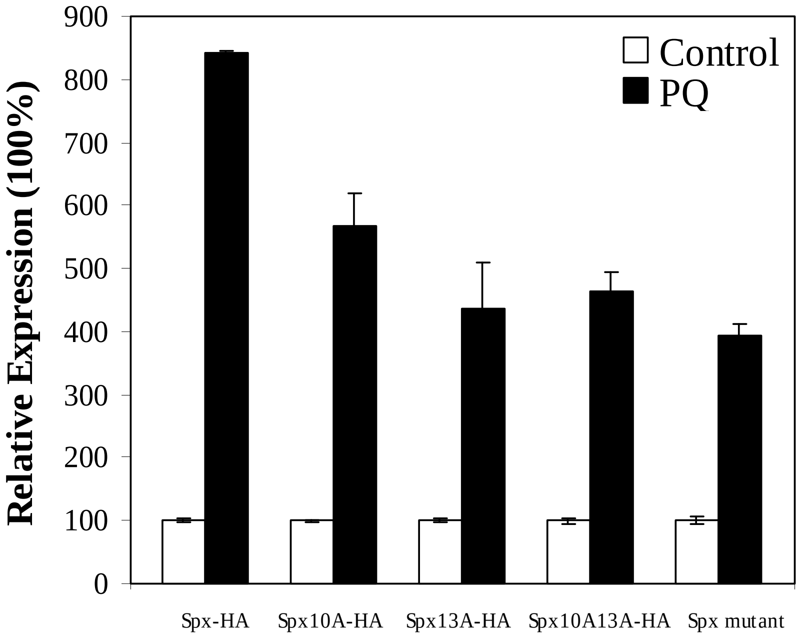

Relative Expression (100%)
900
800
700
600
500
400
300
200
100
0
Control
PQ
Spx-HA
Spx10A-HA
Spx13A-HA
Spx10A13A-HA
Spx mutant
